# Tree Growth and Climate Relationship: Dynamics of Scots Pine (*Pinus Sylvestris* L.) Growing in the Near-Source Region of the Combined Heat and Power Plant During the Development of the Pro-Ecological Strategy in Poland

**DOI:** 10.1007/s11270-015-2477-4

**Published:** 2015-06-22

**Authors:** Barbara Sensuła, Sławomir Wilczyński, Magdalena Opała

**Affiliations:** Institute of Physics-Center for Science and Education, Silesian University of Technology, Konarskiego 22B, 44-100 Gliwice, Poland; Institute of Forest Ecosystem Protection, Department of Forest Protection, Entomology and Forest Climatology, University of Agriculture in Krakow, Al. 29 Listopada 46, 31-425 Kraków, Poland; Department of Climatology Faculty of Earth Sciences, University of Silesia, Bedzinska 60, 21-200 Sosnowiec, Poland

**Keywords:** Pine, Climate changes, Pollution, Tree growth, Combined heat and power station, Carbon isotopes in needles

## Abstract

Since the 1990s, the emission of pollutants was reduced in a majority of Polish and developing country factories whereas the level of energy production was similar to that prior to the 1990s. The conifer investigated in this study has grown for many years under the stress of industrial pollution. Despite this, the trees are preserved, to a large extent, sensitive to the natural climatic factors. We present a complex analysis of the climatic (sunshine, temperature, precipitation, humidity, and wind circulation) and anthropogenic factors influencing the radial increment dynamics of Scots pine (*Pinus sylvestris* L.) growing in the vicinity of the combined heat and power station in Łaziska (Poland). We analyzed the spatiotemporal distribution of growth reductions, the depth of reduction with respect to the distance from the emitter, the relationship between tree growth and climate during the industry development period and during proecological strategy application . Samples of carbon isotopic composition in pine needles from 2012 to 2013 were additionally determined. Pines series of 3 positions indicate that they have a similar sensitivity to most climatic elements of the previous and given year, but there is also a different rhythm between the studied populations of incremental growth of pines. The causes of diversity are due to the different types of habitat (site types) and industrial pollution. The variation in carbon stable isotopic composition in pine needles was connected with an increase of CO_2_.

## Introduction

Trees are a very good archive of ecosystem change in which they are grown. Trees are sensitive to climate changes and anthropogenic pollution. The changes in the ecosystem can disturb the metabolism and physiological processes of trees, and consequently, they also have an effect on the wood structure (Schweingruber 1996; De Vries et al. 2000). Climatic and anthropogenic signals can be recorded in tree ring width (TRW) and the isotopic composition of wood and its components (Schweingruber 1996; De Vries et al. 2000; McCarroll et al. 2009; Sensuła et al. 2011; Sensuła and Pazdur 2013a, b ; Pazdur et al. 2007, 2013). The size of TRW reduction can be affected by the chemical composition of pollution and also depends on the distance from the pollution source (e.g., Wilczyński 2006; Elling et al. 2009). Therefore, the annual TRW reduction can be a source of information about the industrial history and changes in the quality of the natural environment (Cook and Innes 1989).

Tree rings are a useful tool in long-term biomonitoring that can provide annual records of ecosystem changes over several decades, even countries. The systematic long-term monitoring of air pollutants is generally limited to the last decadas and restricted to the point source (e.g., Szychowska-Krąpiec and Wiśniowski 1996; Malik et al. 2012). Trees are an archive of historical changes in the environment. For years, dendrochronological monitoring has successfully been used in the studies of industrial development involving different types of industrial production, such as power stations (e.g., Levanič and Slapnik 2006). Abrupt changes in environmental conditions, such as an increase in air pollution, influence cambium activity and can be responsible for the occurrence of abrupt growth reductions or missing rings (Schweingruber 1986). Until the time that human activities began to alter the natural cycle of different elements carbon dioxide, nitrogen oxides, and sulfur oxides served as one of the major limiting factors that controlled the dynamics, biodiversity, and functioning of many ecosystems (Vitousek et al. 1997). Some of the pollutants are restricted to small, near source region whereas the others are distributed over much larger areas. Human alterations of the carbon, nitrogen, and sulfur cycle have accelerated losses of biological diversity, especially among sensitive plants, and subsequently, the animals and microbes that depend on these plants. It has also caused changes in the plant and animal life and ecological processes. Nitric oxide plays several critical roles in atmospheric chemistry, including catalyzing the formation of photochemical (or brown) smog. In the presence of sunlight, nitric oxide and oxygen react with hydrocarbons emitted by automobile exhausts to form ozone, the most dangerous component of smog. Ground-level ozone has serious detrimental effects on human health as well as the health and productivity of crops and forests. Nitric oxide, along with other oxides of nitrogen and sulfur, can be transformed in the atmosphere into nitric acid and sulfuric acid, which are the major components of acid rain (Vitousek et al. 1997).

The stable isotope ratios of the light elements (carbon, oxygen, and hydrogen) in the plant are very valuable tools in the investigating climate changes and anthropogenic effects connected with the development of industry. The observed anthropogenic impact of the global carbon cycle, mainly related to fossil fuel and biomass burning, land-use changes, and various industrial activities (O’Leary 1981; Leavitt and Long 1982) causes change in the isotopic composition of carbon not only in the atmosphere but also in the biosphere. Nevertheless, there is lack of short-term contemporary spatiotemporal analysis of the current fractionation of carbon isotopes within an industrial area, in near source region.

The investigated area is located near to combined heat and power plant “Łaziska,” which is the third in Silesia and the ninth largest power station in Poland and produces about 4.2 % of the nation’s electricity production. In 2000, the Łaziska Power Station became part of Polish Energy Company (PKE). Since 2011, it belongs to the Tauron Wytwarzanie SA. The power plant in Łaziska was founded in 1917. In the years 1927–1953, it was the largest power plant in Poland. According to the assumptions of the investor, the power plant was to be powered by coal of inferior quality, the large quantities of which were difficult to sell. In 1953, the power of Łaziska was 191.1 MW. Since 1960, electricity production has quadrupled. The next stage of expansion of the power plant included major investments in environmental protection: the creation of a sewage treatment plant, installation of electrostatic precipitators on new blocks, electricity produced exclusively on new blocks. In 1990, the Łaziska Power Station was one of the most oppressive environment. In 1990, the Łaziska Power Station included provisions contained in the Decision Restorative Department of Environmental Protection, Water and Geology Provincial Office in Katowice. Since that time, boiler furnace systems for NOx reduction were modernized, and also, flue gas desulfurization plant was modernized. In fact, in 2000, the power station disappeared from the list of companies most burdensome for the environment, and today, this power plant is one of the best, the most environmentally friendly, and the most modern coal-fired power plant in Poland. Figure 2 shows the effect of the power plant modernization and the changes in level of pollutant emission, whereas the level of energy production is similar. Decreases in the high rate of air pollution in 1990s clearly reflect the successive stages of the modernization of the industrial plant; at the same time, it can be observed that the level of production at that time remains the same. Most of the modernization in different plants and the industrial sector in Eastern Europe is connected with access to EU funding in the past decades and EU legislation and the implementation of restrictive governmental regulations on emissions.

**Fig. 1:**
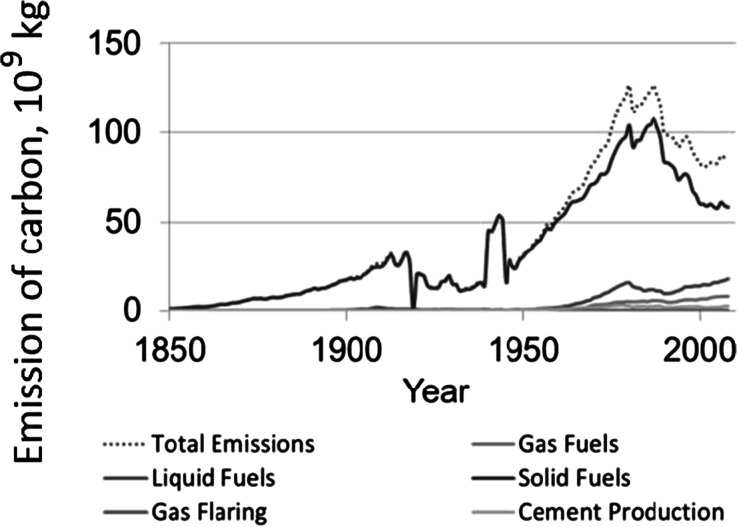
National level of carbon emissions from different sources from the 1850–2012 period. The emission of carbon is expressed in million metric ton of carbon

**Fig. 2:**
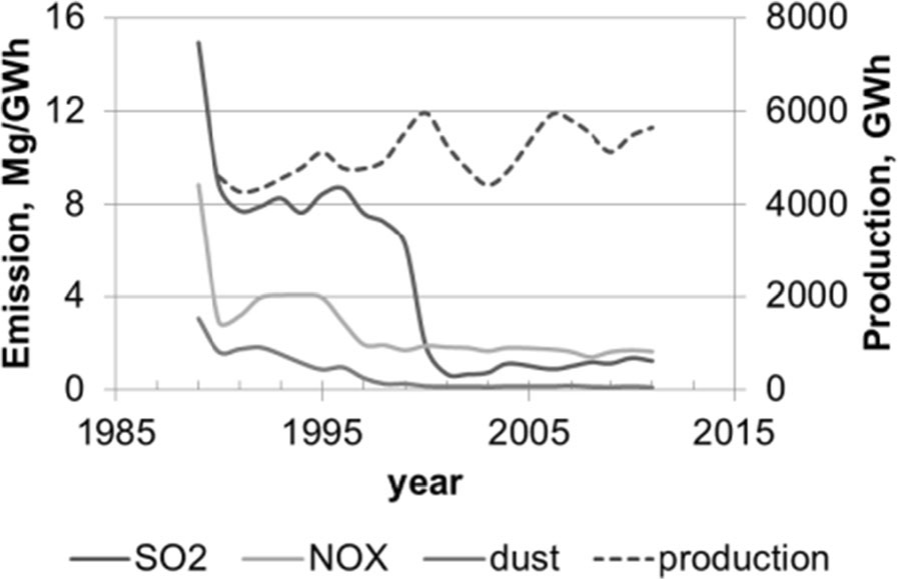
Emissions of SO_2_, NO_x_, and dusts during production of energy by Combined Heat and Power Plant in Łaziska (Poland) from 1989 to 2012

The investigated area is characterized by very significant transformation of the natural environment caused by mining and metallurgy, as well as the growth of Poland’s largest urban-industrial agglomeration (Kondracki 2001). The relief of the Katowice Upland is one of the most transformed in Poland and Central Europe. Typical anthropogenic landforms, such as incisions, embankments, excavations, dumps, water reservoirs, and water flows, commonly occur (Szypuła 2014). Changes in the air quality (high amount of dust and gas pollutants within the entire area of the Katowice Region, contributing to the occurrence of “acid rains” phenomenon) were reported (Leśniok 2011Environmental pollution adversely affects the health of the population (Absalon and Ślesak 2010), quality of life (Absalon and Ślesak 2012), as well as the condition of forests (Breymeyer 1998) and soil properties (Heller et al. 1998). The predominant types of soils in this area are *Albic Luvisol. Haplic Podzols* are present in the flat depressions and *Haplic Fluviosols* in the valley bottoms. Technic *Regosols* and different soil units of contaminated soils are typical for the urban-industrial areas (Lazar 1962). Potential natural vegetation is composed of mixed oak-pine forests (*Querco-Pinetum*) and oak-hornbeam forests (*Tilio-Carpinetum*) (Matuszkiewicz 2008). At present, acidophilus oak forests (*Quercetea robori-petraeae*) are fragmentarily preserved, although the artificial planting of pine predominates. This area is characterized by the highest level of industrialization in Poland (Figs. 1 and 2, Marland et al. 2008), where the highest values of dust and gaseous pollutants were recorded in late 1970s. It should be mentioned that other emitters from the southern part of Poland and from Germany and Tcheck Republic could influence not only the atmosphere but also the biosphere and trees.

The aim of the presented study is the analysis of the factors influencing the radial increment dynamics of Scots pine (*Pinus sylvestris* L.) growing in the vicinity of the Combined Heat and Power station (CHP) in Łaziska (Poland). The specific objective of this study was the determination of CHP air pollution effects on the following: (1) the spatiotemporal distribution of growth reductions, (2) the depth of reduction with respect to distance from the emitter, (3) relationship between radial growth of pines and climate during the period of development of industry, and (4) the spatiotemporal fractionation of carbon stable isotopes in the needles of pine collected in 2 consecutive years (2012 and 2013).

## Materials and Methods

**Fig. 3:**
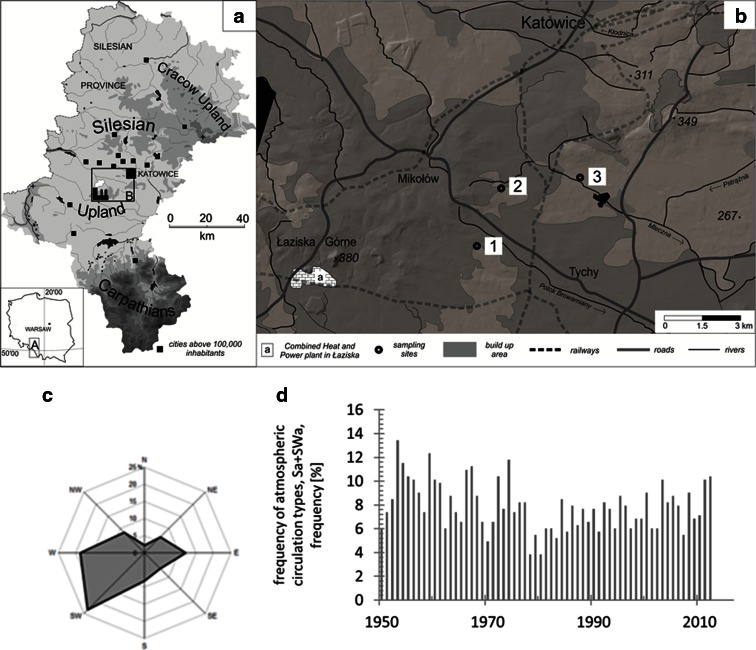
Investigated site: **a** location of the study area in the Silesian Province; **b** detailed location of the sampling sites (1–3) in the vicinity of the Combined Heat and Power Plant in Łaziska; **c** distribution of wind directions; and **d** dominant circulation types

The sampling sites are exposed to typical anthropogenic stress of heavily urbanized areas in close to proximity of the large power station in Łaziska. The highest part of the study area reaches about 350 m a.s.l. at Mikołów Horst, but the elevation near the research sites goes down to 260–290 m a.s.l. Three sampling sites located in ENE direction from the emitter were selected (Fig. 3a, b) in accordance with the direction of the prevailing wind (Fig. 3c), at distances from 3 to 12 km from the plant (Table 1). The scale of impacts of regional and local types of pollution can be dependent on the meteorological and circulation conditions. The circulation type can significantly influence the pollution level and favor the concentration or dispersion of air pollution (Niedźwiedź and Ustrnul 1989). Among all synoptic situations, the highest pollution levels (dust, SO_2_, NO_2_, and O_3_) occurred during anticyclonic circulations, with an advection of air masses from the south and southwest (Sa and SWa) (Leśniok et al. 2010).

**Table 1:** Main characteristics of the sampling sites: time span, number of cores collected, and localization of sampling site

Research site	Lab code	Time span (years)	No of cores	Distance from pollution source [km]	Geographical coordinates	Elevation [m a.s.l.]
1	LW	1922-2012 (91)	20	3	50° 08′ N, 18° 56′ E	280
2	LM	1914-2012 (99)	20	7	50° 09′ N, 18° 57′ E	290
3	LP	1917-2012 (96)	20	12	50° 10′ N, 18° 59′ E	260

Despite the high building density, uneven distribution of forests, and their high species and age differentiation, sites with similar habitat conditions and age structure have been selected. Scots pine (*P. sylvestris* L.) at an age of about 100 years predominated at all the research sites. Due to the species composition of the forest in the study area, Scots pines were selected for investigations. This common species is considered to be sensitive to the anthropogenic effect (Schweingruber 1996; De Vries et al. 2000; Sensuła et al. 2011; Sensuła and Pazdur 2013a, b; Pazdur et al. 2007, 2013). In order to avoid a different dendroecological reaction of juvenile wood, an attempt was made to select pine stands aged between 90 and 100 years (felling age of Scots pine). In summer 2012, cores of the dominant trees were taken with Pressler’s borer at the breast height. The conifers investigated in this study covered the time span from 1914 to 2012. The time span of the developed chronologies ranges from 91 to 99 years (Table 1).

The TRW of all samples were measured within an accuracy of 0.01 mm with the use of the LINTAB 6 device with a microscope and TSAPWin software (Rinn 2010). The accuracy of tree ring dating the rings was verified by the COFECHA program (Holmes 1983). The normalization of the TRW series was carried out using the ARSTAN computer software (Cook and Holmes 1999). For each year, the values of ring width indices of the residual series were averaged, so that the residual chronology was created for each pines population.

The chronologies for the 1951–2012 period were tested with the between-tree signal (rbt), the expressed population signal (EPS), and the signal to noise ratio (SNR). Rbt is defined as the mean interseries correlation calculated between all pairs of indexed series drawn from different pines. Rbt is commonly used to represent the chronology signal (Briffa and Jones 1992). The EPS is a statistic for examining the common variability in a chronology, and it is dependent upon the sample depth, whereas the SNR informs about the ratio between the signal (short-term variation) and the noises (long-term variation) contained in chronologies of the investigated trees (Wigley et al. 1984).

**Fig. 4:**
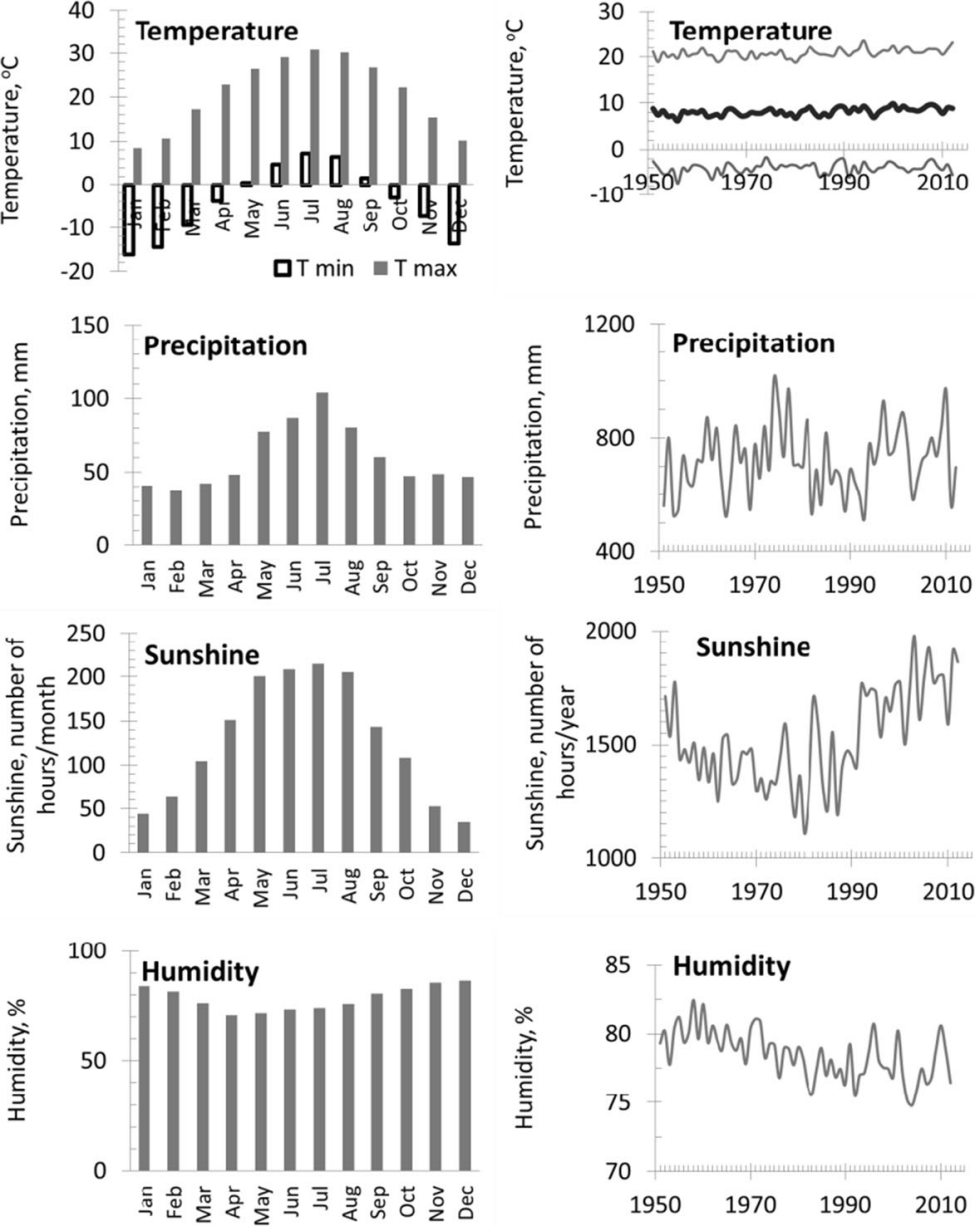
Climatic conditions in investigated areas from 1951 to 2012: (temperature: monthly minimum and maximum, mean annual; precipitation: monthly and annual sum, sunshine monthly and annual sum of number of hours, humidity monthly and annual mean) for the Katowice meteorological station from 1951 to 2012

A value of 0.85, usually cited as an acceptable threshold, was also used here (Wigley et al. 1984; Speer 2010). Climate data (monthly air temperature, total precipitation, humidity, sunshine, and wind directions) from the nearest meteorological station located in Katowice (Fig. 4). The meteorological data covered the period of time since 1951–2012 were obtained thanks to the Polish Institute of Meteorology and Water Management (IMGW-PIB).

During the 1951–2012 period, the investigated area was characterized by a mean annual temperature ca. 8 °C, the mean total annual precipitation equal to ca. 720 mm/year, sunshine equal to ca. 1530 h/year, and relative humidity equal to ca. 78 %. The study area was dominated by winds from the southwest and the west (Fig. 3c). The mean annual temperature and sunshine increased, whereas humidity decreased during the last decades. The highest temperature, sunshine, and precipitation were noted between May and August. A significant variation of climatic factors has influenced tree growth and the dynamics of tree ring radial growth. Climatic factors affecting the radial growth variability were determined by the correlation and response functions (Fritts et al. 1971; Fritts 1976). The correlation and response coefficients between site residual chronologies and monthly climatic data were calculated by the RESPO computer software (Holmes and Lough 1999) and then compared with the results from Dencdroclim2000 (Biondi and Waikul 2004). This program uses 1000 bootstrapped samples to compute the correlation and response coefficients and to test their significance at the 0.05 level. The dependent variables were the indices of site residual chronology from 1951 to 2012 (*n* = 62). The independent variables were the monthly values of a maximum and minimum temperature, total precipitation, total sunshine duration, and relative humidity from August of the year previous to September of the current year of the ring formation (*n* = 14). A principal component analysis (PCA) was applied to describe the relationships between the radial increment of pines and climate parameters and to identify the factors affecting TRW. PCA was used to reduce the residual series of 60 trees to orthogonal principal components. Based on a screen plot, two first principal components were used. The identification of PC1 and PC2 was based on an analysis of the component scores. For this purpose, the correlation and response function were again used. The dependent variables were the scores of PC1 and PC2.

Air pollution emissions were not continually monitored in the investigated area, and most of the data is only available for the last 20 years. We used the following database of pollution: CO_2_ emission in Poland since 1950—Carbon Dioxide Information Analysis Center (Marland et al. 2008); CO, SO_2_, NO_x_, and dust emission in the Silesian Province since 1975—Statistical Yearbooks of the Environmental Protection 1975–2012, Reports of Environment Protection Bureau in Huta Katowice (B.O.Ś 2002, 2006); SO_2_, NOx, and dust emission from Combined Heat and Power Plant in Łaziska—Environmental Statement of Łaziska Power Station 2009, 2012). Due to the administrative changes in Poland in 1999, data for the Province for the years 1975–1999 was calculated as a sum of values for three former provinces: Bielsko, Katowice, and Czestochowa Province and data for current Silesian Province was used for the years 2000–2012. The correlation between local air pollution emission on TRW was made with using Statistica softwere (Statistica 10.0 software).

An additional dendro-ecological analysis was carried out according to the method used by Schweingruber (1986), in which the reduction values are calculated as the total TRW in the reduction period in relation to the same number of rings from the period preceding the reduction. The calculated reductions were classified as follows: moderate reductions, 30–50 %; strong reductions, 51–70 %; and very strong reductions, >70 %.

Additionally, the samples of pine needles from each site were collected in 2012 and 2013. The samples were washed in distilled water and then dried and homogenized and shredded. The measurement of carbon isotopes was carried out in the Mass Spectrometry Laboratory (Department of Radioisotopes, Institute of Physics, Gliwice, Poland), using the isotope ratio mass spectrometer (IsoPrime) coupled to the Eurovector elemental analyzer at a combustion temperature of 1020 °C (GV Instruments, Manchester, UK). The precision on triplicates was ±0.26 ‰ (*n* = 50). The relative deviation of the isotopic composition is expressed in parts per thousand (‰), as follows:

$$ \delta =\left(\frac{R_{sample}}{R_{s \tan dard}}-1\right)\cdot 1000 $$

where R_sample_ and R_standard_ are the ratios of the heavy to the light isotope concentration in the sample and in the standard, respectively. The reference standard for carbon is the isotopic ratio in the Vienna Peedee Belemnite (VPDB).

## Results

**Fig. 5:**
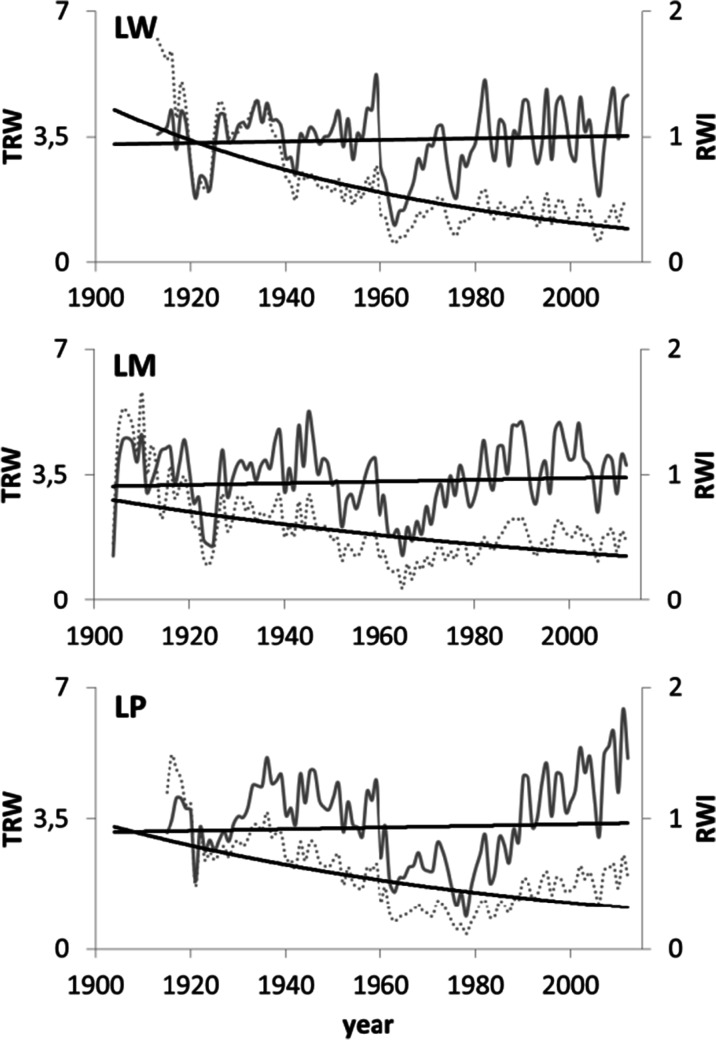
Annual increment growth of Scots pines from the investigated sites and the negative exponential fit lines (*solid lines* RWI, *pointed lines* TRW)

**Fig. 6:**
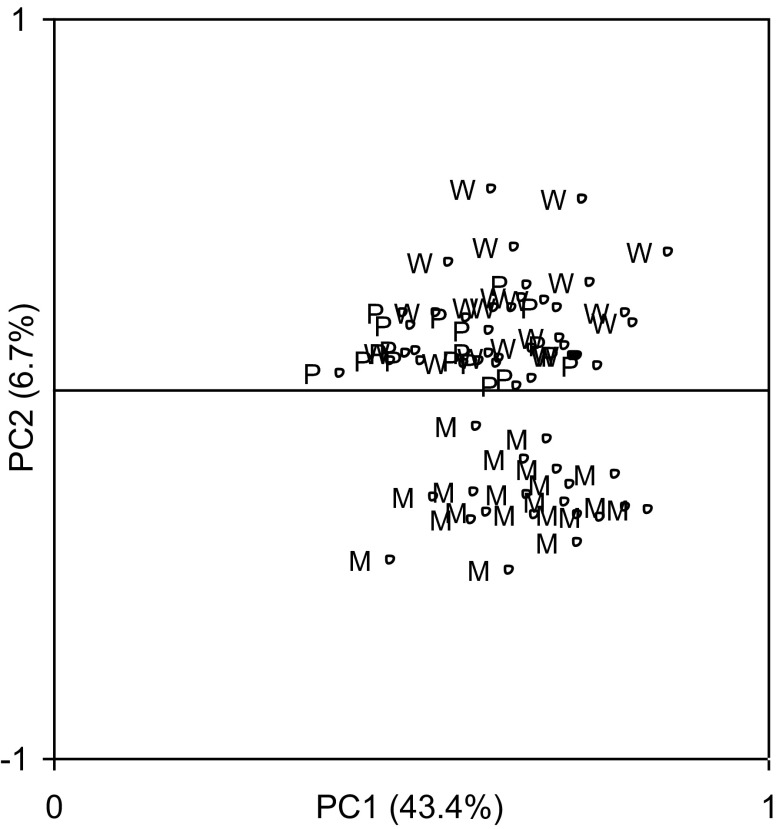
The location of the residual series in relation to the component loadings of PC1 and PC2. *The brackets* contain the variance of the chronologies explained by PC1 and PC2. *M* population LM, *W* population LW, and *P* population LP

**Fig. 7:**
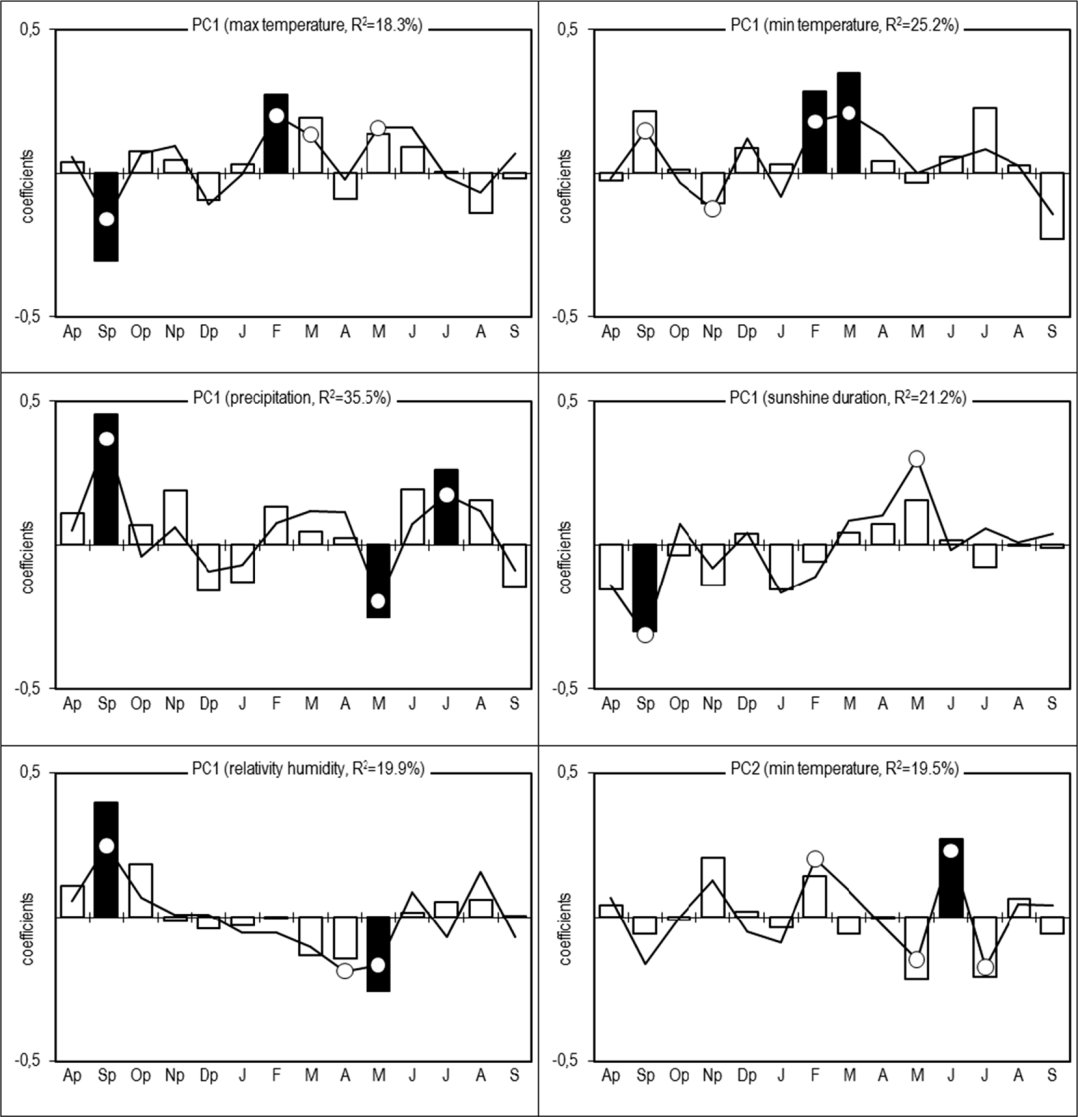
Correlation (*bars*) and response function (*lines*) coefficients between the scores of PC1 and PC2, and the monthly maximum and minimum temperatures, total precipitation and sunshine duration, and relative humidity for all months from the previous August (Ap) to the current September (S) from 1951 to 2012. Significant values (at the 95 % confidence limit)—*black bars* and *white dots*, *R* ^2^—the coefficient of multiple determination

**Fig. 8:**
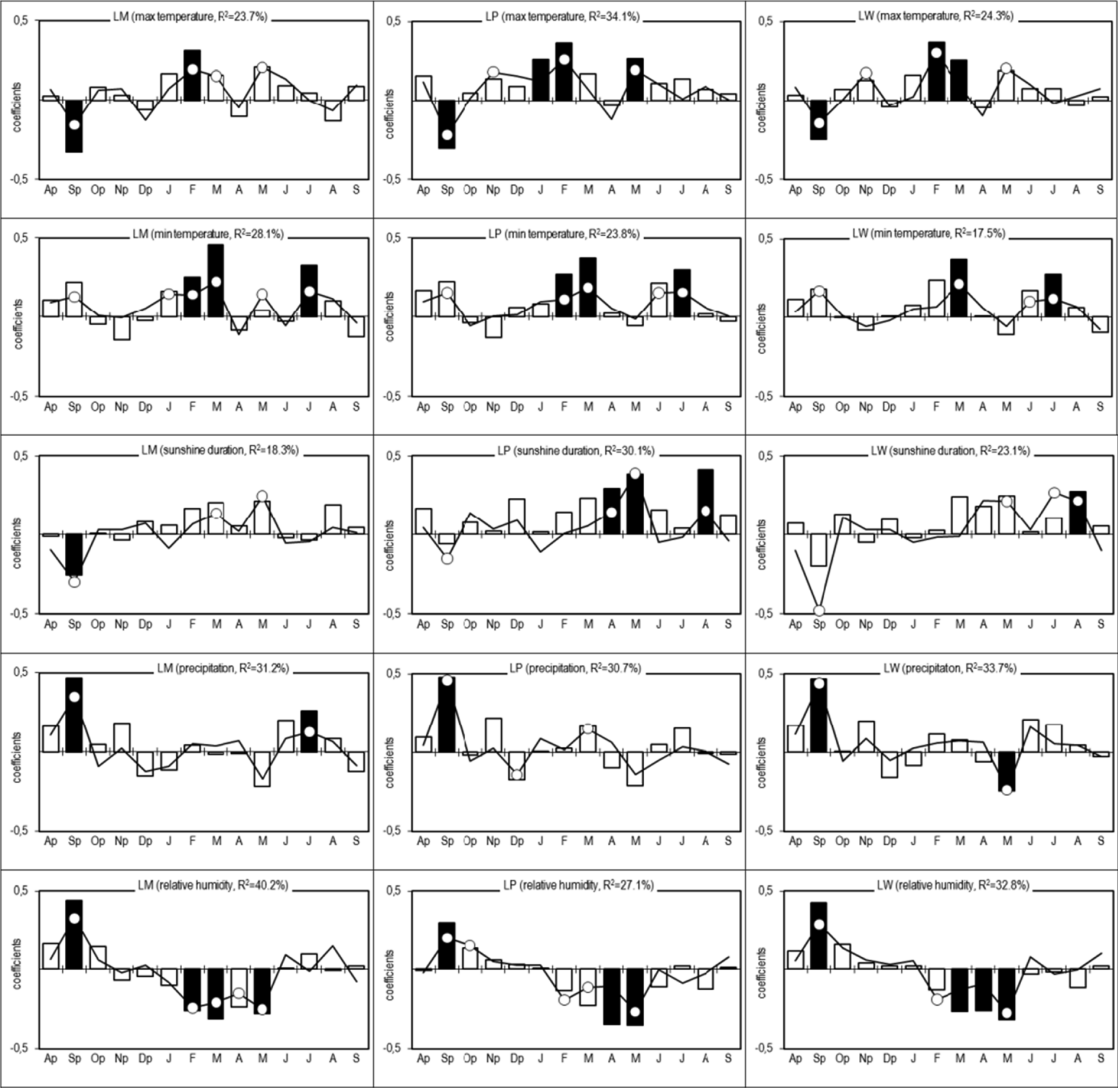
Correlation (*bars*) and response function (*line*) coefficients between the site residual chronologies and the monthly maximum and minimum temperatures, total precipitation, sunshine duration, and relative humidity for all months from the previous August (Ap) to the current September (S) from 1951 to 2012. Significant values (at the 95 % confidence limit)—*black bars* and *white dots*, *R* ^2^—the coefficient of multiple determination

The series of TRW and normalized indices (RWI) were created for each tree (Fig. 5). The statistical characteristics of the site residual series and chronologies for the period 1951–2012 are presented in Table 2. The pines of each population are characterized by a high similarity of short-term increment reactions (r_bt_ indices ranged from 0.399 to 0.514) (Table 2). The site residual chronologies are characterized by a high representativeness (indices EPS ranged from 0.930 to 0.954). The percentage of the variance of 60 residual series explained by PC1 is 43.4 % and that by PC2 is 6.7 % (Fig. 6). PC1 is the most effective for the description of the variations in the radial increment. The location of the residual series with regard to the loadings indicates that PC1 integrates the series. The residual series correlate positively with PC1 (*P* < 0.01) (Fig. 6). The PC2 differentiates the series into two groups. The first group is constituted by a series of LW and LP populations. They correlate positively with PC2. The series of the LM population correlate negatively with PC2 (Fig. 6). This division of series is related to the site habitat. As a result, PC1 can be described as a multisite radial increment index. This has a similar effect on the short-term radial increment in all the pine populations. The second principal component can be described as a specific parameter for a tree stand site. PC2 describes factors to which the pines of each population show different sensitivity. The size of the radial increment shows a statistically significant relationship to the maximum temperature of September of the year preceding the radial increment and a maximum temperature in February, March, and May of the current year. The variation of the radial increment is influenced by the September minimum temperature of the previous year and the minimum temperature of February and March of the current year. It is determined that PC1 show a significant relationship to the total hours of sunshine during September of the previous year and May of the year of the ring formation (Fig. 7). Moreover, it is significantly correlated with the total precipitation of the previous September and May, and July of the current year. The PC1 also show a significant relationship to the mean relative humidity of the previous September and the current April and May (Fig. 7). The PC2 indicate a significant relationship to the minimum temperature of February, May, June, and July in the year of the tree ring formation (Fig. 7). The analyses of the correlation and response functions undertaken for 3 site chronologies confirm the above results (Fig. 8). All the elements included in the analysis of climate variability affect the dynamics of radial growth. This is evidenced by the high values of the coefficients of determination. Comparing two different softwares—RESPO and Dendroclim2000—it is evident that both software solutions gave similar selected statistics results at the 95 % confidence limit. In figures, we presented the RESPO results.

**Table 2:** Statistical characteristics of the site residual series and chronologies for the 1951–2012 period: number of series investigated, mean tree ring width (TRW), mean tree ring indices (TRI), mean sensitivity, between-tree signal (r_bt_), expressed population signal (EPS), signal to noise ratio (SNR)

Lab code	No of series	Mean TRW (mm)	Mean TRI	Mean sensitivity	Correlation with master chronology	R_bt_	EPS	SNR
LW	20	1.95	0.97	0.284	0.578	0.508	0.953	20.6
LM	20	2.07	0.99	0.308	0.520	0.514	0.954	21.1
LP	20	1.98	1.00	0.285	0.591	0.399	0.930	13.3

**Fig. 9:**
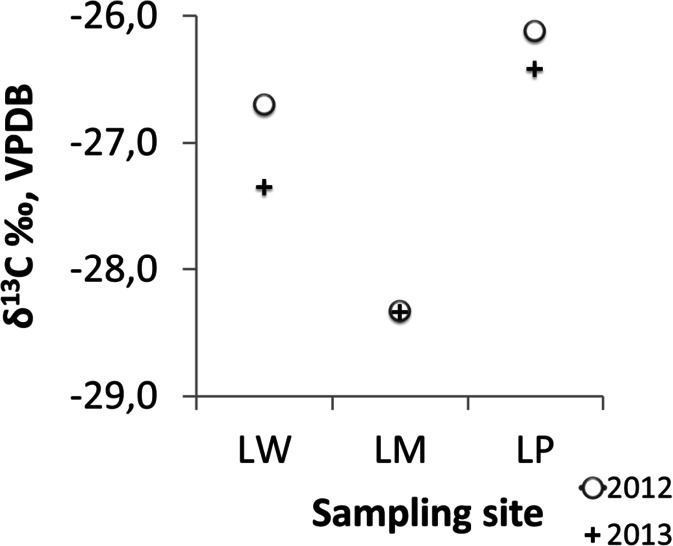
Stable carbon isotope fractionation in pine needles, collected in 2012 and 2013 from investigated sites

**Fig. 10:**
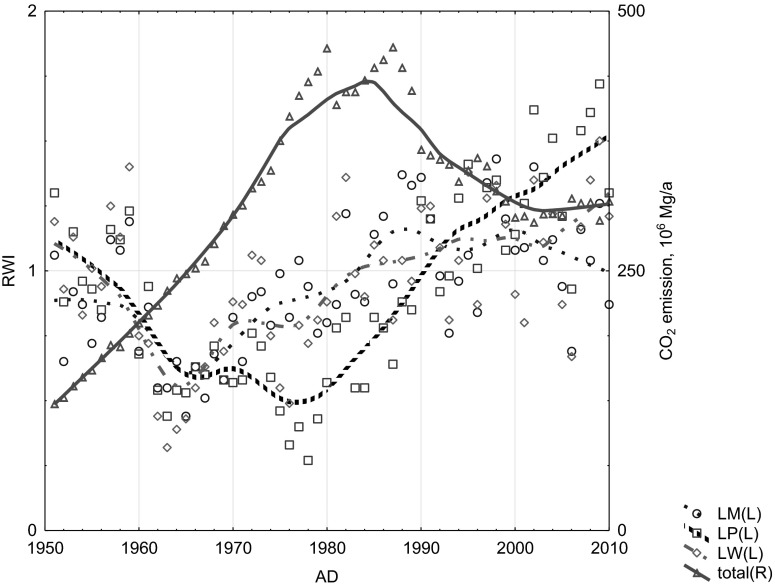
Effect of variation of CO_2_ emissions from fossil fuel combustion in Poland on the dynamics of pine radial growth (locally weighted scatter plot smoothing)

**Fig. 11:**
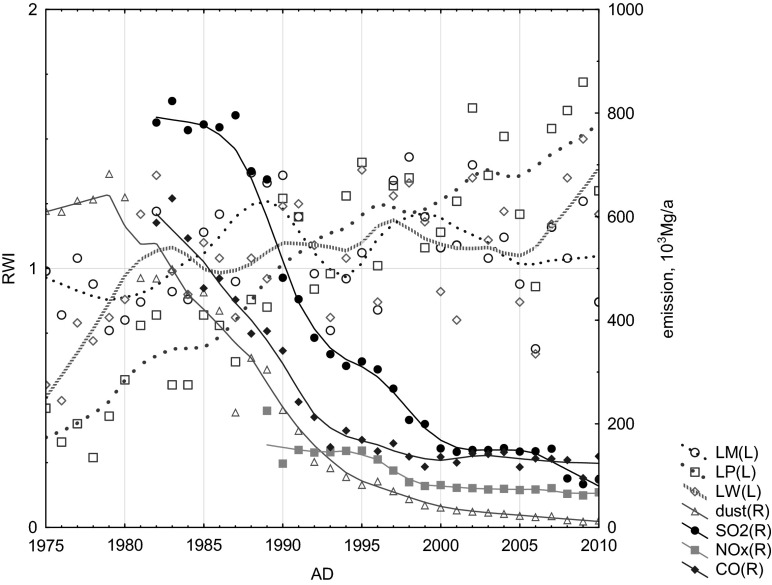
Effect of the decrease of local air pollution emissions (CO, SO_2_, NOx, dusts) in the Silesian Province on the dynamics of pine radial growth (locally weighted scatter plot smoothing)

**Fig. 12:**
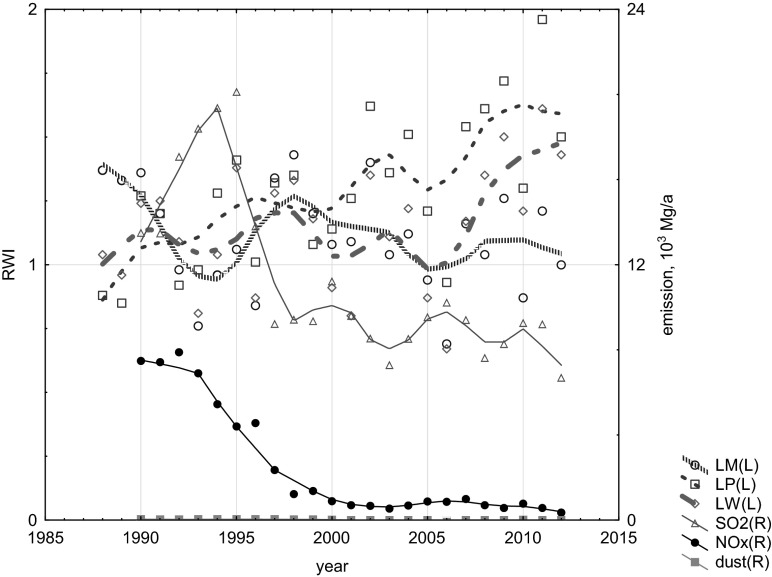
Effect of the decrease of local air pollution emissions (CO, SO_2_, NOx, dusts) in the Combined Heat and Power Plant in Łaziska on the dynamics of pine radial growth (locally weighted scatter plot smoothing)

The analysis of the influence of local air pollution emission on TRW confirmed the regional (national) negative effect of the increase of CO_2_ emissions from fossil fuel combustion (Figs. 9 and 10), the negative effect of increase of the local (Silesian, Fig. 11) air pollution emissions (CO, SO_2_, NO_x_, dusts) and also the local negative effect of the distribution of air pollution such as NOx, SOx, and dusts from sources near the investigated site—the Combined Heat and Power Plant in Łaziska (Fig. 12). The signal strength is variable and depends on the distance from the source. The reduction of gaseous and dusts emission is also recorded in the TRW.

The spaciotemporal analysis of industrial air pollution, such as sulfur dioxide, nitrogen oxides, and dust, emitted from the Łaziska Power Station shows a significant linear correlation between the emission of NO_x_ pollution and TRW between 1989 and 2000 only in one sampling site (LM) located at a distance of 7 km from this near source of pollution (*r* = −0.68, *p* < 0.02). Higher emissions of NOx reduced TRW. Due to the lack of data, it is impossible to compare the pollution level before 1989 and TRW reduction. Between 1990 and 2000, this power station was placed on the list of the most oppressive for environment. Since 2000, there has been significant linear correlations between dusts and NO_x_ emitted from the Łaziska Power Station and TRW observed for the sampling site located at a distance of 3 km (*r* = −0.55 for NO_x_ and *r* = −0.53 for dust emission, *p* < 0.1, respectively) and at a distance of 12 km from the power station (*r* = −0.51, for dust emission, *p* < 0.1). Increase of SO_2_ emission corresponds to narrow rings, and a decrease of SO_2_ emission corresponds to the increase in TRW.

We have also noted differences in carbon stable isotope fractionation in pine needles (Fig. 9), which were similar for the LP and LW, but different in the LM.

## Discussion

The three tree ring chronologies built in this study create the local pattern of each pine population undertaken in this study. These patterns are the archive of the pine incremental response to various environmental factors. A strong positive correlation with the first principal component of pines series from 3 sampling sites indicates that they have a similar sensitivity to most climatic elements described by PC1. A scatter of results of test batches in relation to PC2 indicates, however, that there are also different rhythms between the studied populations of incremental pines. Therefore, climate-growth relationships estimated for the LM population should not be generalized to other populations. The cause of the diversity of these compounds may be the impact on tree industrial pollution (Wilczyński 2006). LM population pines showed smaller radial growth reductions, as compared to others. It seems that in this case, a type of habitat (site type) where tree has been growing is significant. The other two populations have been growing in the poorer habitats of a mixed coniferous forest site type. It is known that the site type has an influence on the incremental rhythm of the trees (Friedrichs et al. 2009; Cedro and Lamentowicz 2011; Dauskane et al. 2011; Wilczyński and Kulej 2013). This most probably resulted in the incremental index series of the pines from the position of the LM was different. We also noted a significantly lower value of δ^13^C (Fig. 9) for the LM population. The isotopic composition of pine needles in this investigated site was similar in 2012 and 2013, whereas the δ^13^C values in samples collected in 2013 were lower than in the samples collected in 2012 in LW and LP population. The decrease of δ^13^C corresponds to the increase of CO_2_ emission to the atmosphere (Pazdur et al. 2007; McCarroll et al. 2009; Keeling et al. 2010; Sensuła et al. 2011; Sensuła and Pazdur 2013a, b).

Human alterations of the carbon, nitrogen, and sulfur cycle have influenced the dynamics, biodiversity, and functioning of many ecosystems and ecological processes. Abrupt changes in environmental conditions, such as increase in air pollution, can be responsible for the occurrence of abrupt growth reductions or missing rings and also for differences in isotopic fractionation (Schweingruber 1986, 1996). A few dendrochronological analysis in association with the isotopic analysis has shown that the long-term modification of the TRW and also carbon isotopic values are indeed modulated by environmental conditions; these conditions could be the following: climate, tree diseases, land use or air quality, as trees absorb natural and anthropogenic compounds (O'Leary 1981; Leavitt and Long 1982; Guerrieri et al. 2009). The analysis of the influence of local air pollution emission on tree rings confirmed the regional (national) negative effect of the increase of CO_2_ emissions from fossil fuel combustion the negative effect of increase of the local air pollution emissions (CO, SO_2_, NO_x_, dusts) and also the local negative effect of the distribution of air pollution such as NOx, SOx, and dusts from sources near the investigated site—the Combined Heat and Power Plant in Łaziska. The signal strength is variable and depends on the distance from the source. The reduction of emission of gaseous and dusts emission is also recorded in the TRW. Tree population analyses under these studies were characterized by the varying r_bt_ value. The highest r_bt_ is noted for the LM population, characterized by the lowest damage (the level of reduction and the number of trees with reductions). On the other hand, the LP population has the lowest r_bt_, this population was characterized by the greatest damage (level of reductions and the number of trees with reductions) (see Table 3). The EPS values of each series significantly exceeded the value of 0.85; therefore, each of the chronologies are characterized by high representativeness. Different climatic factors occurring in the previous and current year had an impact on the variability of the radial growth of the investigated pines. The results of the analyses of climate-growth relationships for 3 populations were similar. This impact was determined by the correlation and response functions, indicating the significance of both types of coefficients (correlation and multiple regression coefficients). It is difficult to clearly identify to which one of the climatic factors the pines from all sites show a similar sensitivity toward, and toward which ones they show a different sensitivity. High values of the determination coefficients indicate a high sensitivity of the pines on the climatic factor, despite the fact that they were exposed to the pressure of industrial pollution. The analysis of variance is often helpful in this type of assessment (analysis) (Carrer and Urbinati 2004, 2006; Yu et al. 2008; Wu et al. 2013). It also applies principal component analysis by identifying the principal separated individual integral components (Wilczyński and Kulej 2013). The correlation and response function analysis performed for PC1 and PC2 help to identify climatic factors described by the two main components. The impact of weather conditions of the previous year on the growth of the current year is evident (Vaganov 1990; Richter et al. 1991; Lindholm et al. 1996; Spurk 1997;Wilczyński and Skrzyszewski 2002; Helama et al. 2013; Juknys et al. 2014). Our results indicate that if there was significant precipitation in the previous year and the weather conditions were cloudy, humid, and cool, but not cold in September, these conditions were favorable to the radial growth increase in the following year. This is due to the fact that the autumn weather conditions affect the tying buds, which are reflected in the growth of pines in the following year. The amount of generated buds is determined by the abundance of flowering in the next year. (La Bastide and Van Vredenburch 1970; Fober 1976; Hejnowicz 1982). Profuse flowering in turn has a negative impact on the tree radial growth (Eis et al. 1965; Chałupka et al. 1976). High temperatures and low humidity in autumn have a positive effect on the flowering of female flowers in the subsequent year. This is evidenced by the results of several studies (Chirov 1964; Andersson 1965; Fober 1976). In turn, cloudy, wet weather with plenty of rain in autumn adversely affect the creation of the buds of flowers (buds) female, preferably donuts and vegetative organs (needles and shoots) (Hejnowicz 1982). These conclusions are supported by the results of our research. Warm and short winters with low humidity positively affect the pines’ growth condition, in effect, the physiological processes that led to the earlier cambium divisions in the trees. A similar effect is obtained in the conditions of a dry, sunny, and warm spring. High temperatures in early spring result in the first division in the pine cambium occurring in April (Schober 1951; Ermich 1959). Many researchers reported a positive impact of high temperatures in late winter on the radial growth of the pines (Richter et al. 1991; Spurk 1997; Wilczyński and Skrzyszewski 2002;Juknys et al. 2014). It should be mentioned that in our studies, we found out positive impact of low humidity and of sunshine in the end of the winter and during spring, on the growth of the pines. Such conditions favor the activation of the cambium and enhance the transpiration of trees, and in effect the increasing of the cambium division. Spurk (1997) points to the positive effects of high humidity on the growth of Scots pine. The relatively high values of determination coefficients, which describe the impact of rain on the growth of the pines, indicate the important role of rainfall in September of the previous year. Low rainfall in May of the current year, which is related to the known positive effects of sunshine on the trees and the low humidity in this month, was also essential for the growth of pines. Abundant rainfall in July of current year had a positive impact on the wood cell formation. This weather condition is conducive to the intense divisions of vascular cambium. Low rainfall during the summer is often a limiting factor for growth pine (Lindholm et al. 1996, 1997; Irvine et al. 1998; Cinnirella et al. 2002; Wilczyński and Skrzyszewski 2002; Tuovinen 2005; Pilcher and Oberhuber 2007; Piovesan et al. 2008; Gruber et al. 2010). It also happens that excess rainfall harms trees, mainly in humid positions (Helama et al. 2013). A shortage of water in the soil when the tree is simultaneously exposed to high temperatures has a particularly negative impact on the tree. The high temperature of the air increases the transpiration which contributes to the disruption of water management in plants. If there is no relationship between TRW and the climatic factors of September of the current year, this means that the radial increment is finished in August. This confirms the results of Henappl (1965) and Schober (1951). Whereas Ermich (1959) shows that large part of the ring is deposited in many species also including pine in September, in the case of favorable weather conditions.

**Table 3:** The size and extent of the growth reductions: duration of reduction, percentage of trees with reduction, level of reduction, and degree of reduction

Lab code	Duration of reduction	Percentage of trees with reductions	Reduction level [%]	Degree of reduction
LW	1960-1990	70	60	Strong
LM	1961-1980	67	36	Moderate
LP	1962-1989	75	65	Strong

**Fig. 13:**
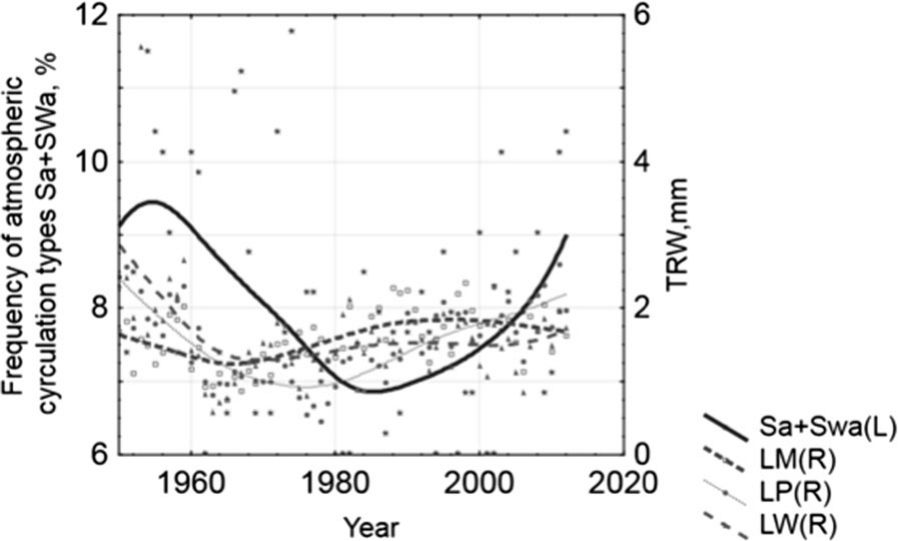
The influence of Sa and SWa circulation on the tree ring width in the 1951–2012 period (weighted least squares plot smoothing)

Tree rings of pines from all the investigated sites showed the influence of air pollution visible as growth suppressions. However, the differences between study sites can be observed in terms of the amount of growth reductions and the number of damaged trees. In general, the pines which were growing near the Combined Heat and Power Plant (CHP) plant in Łaziska produced suppressed annual rings from 1960 to 1990 (Table 3). The existence of the distinct growth reductions in the period from 1960 to 1990 can be confirmed by the results of earlier studies from other parts of the Silesian Upland (e.g., Danek 2007; Malik et al. 2012). As at every research conducted within the major industrial regions, results show not only the record of pollution of a local type, but a record of pollution transferred from the industry located in the entire Upper Silesian Industrial District is also highly probable. Such a situation was described by Danek (2007). Comparison of frequency of Sa and SWa circulation (Niedźwiedź 2013, Fig. 3c) with the TRW shows that there is an inverse relationship between them (Fig. 13). During the period of growth reductions, the higher frequency of this type of circulation can be noticed. This relationship is not linear, the influence of the amount of pollution in the subsequent years is substantial. However, in the absence of pollution measurement data, this additional data may be useful in order to confirm that increased air pollution resulted in growth suppressions in the 1960s, 1970s, and 1980s.

## Conclusions

Dynamics of Scots pine (*P.sylvestris* L.) growing in the near-source region of the Combined Heat and Power Plant Łaziska is the response of trees to various climatic and anthropogenic environmental factors. Pines series of 3 positions indicate that they have a similar sensitivity to most climatic elements of the previous and present year, but there are also different rhythms between the studied population incremental pines. The cause of the diversity may be, for example, the results of a different type of habitat (site type) and industrial pollution. Our results indicate the conditions of year previous to the formation of a new tree ring in the following year that favor the activation of cambium and increase the radial growth of pine, enhance the transpiration of trees, such as (1) abundant precipitation in September of previous year; (2) cloudy, humid, and cool, but not cold autumn; (3) warm and short winter with low humidity. Similarly, effects are provided by the condition of a dry, sunny, and warm spring, or low humidity and lots sunshine at the end of the winter and during spring. Low rainfall in May of the current year, which is related to the known positive effects of sunshine on the trees and the low humidity in this month were also essential for the growth of the pines. Abundant rainfall in July of current year had a positive impact on the wood cell formation. The similarity of the short-term incremental rhythm pines growing in different habitats is a fact, and all the above factors also impacted this. It should be noted that in addition to the common incremental features were also different characteristics of the studied incremental pines populations. It turned out that LM pine population differed from the others only in minimal sensitivity to the temperature in February and in late spring and summer. Thus, habitat conditions associated with fertility positions are modeled on the above relationships. We have also noted differences in carbon stable isotope fractionation, which were similar for the LP and LW population, but different in the LM pine population. The dendroecological analysis showed the influence of air pollution on dynamics of radial pine growth as growth suppressions. In general, the pines which grew near the Combined Heat and Power (CHP) Plant in Łaziska produced suppressed annual rings from 1960 to 1990. The obtained results show that the pine tree ring and needles can be an archive not only of local type pollution records, but also they can be an archive of pollution transferred from the industry located in entire province or country. The multiannual series of the frequency of Sa and SWa circulation compared with tree ring index show that there is an inverse relationship between them. This relationship is not linear. The emissions of SO_2_, NO_x_, CO_2_, and dusts and other pollution lead to serious disturbance in tree metabolism. In effect, pines growing in industrial regions formed reduced rings. Strong growth declines for all of the sites were noted from the 1960s till late 1980s. Since the 1990s, when proecological strategy was developed in the factory, the emission of pollutants reduction whereas the level of energy production was similar as before the 1990s. This reduction of pollution is recorded in the increase of the TRW. Scots pine populations, investigated under this study, have grown for many years under the pressure of industrial pollution. This influence was reflected mainly in the long-term decline in radial growth. Despite this, the trees are preserved to a large extent, sensitive to the natural climatic factors. This sensitivity of pines can be significant in the reconstruction of the climate in the past so as to better understand the future. The analyses of tree ring δ^13^C variations are promising tools for investigating the carbon deposition to forests. Moreover, regional analyses of needles δ^13^C variations could enable the mapping of the impact of carbon deposition on forest ecosystems and in assessing the input of pollutants into plant communities. Such information is strongly needed worldwide.
